# Age‐related changes to macrophages are detrimental to fracture healing in mice

**DOI:** 10.1111/acel.13112

**Published:** 2020-02-25

**Authors:** Daniel Clark, Sloane Brazina, Frank Yang, Diane Hu, Christine L. Hsieh, Erene C. Niemi, Theodore Miclau, Mary C. Nakamura, Ralph Marcucio

**Affiliations:** ^1^ Department of Orthopaedic Surgery School of Medicine Orthopaedic Trauma Institute Zuckerberg San Francisco General Hospital University of California San Francisco San Francisco CA USA; ^2^ Division of Periodontology Department of Orofacial Sciences School of Dentistry University of California San Francisco San Francisco CA USA; ^3^ Division of Rheumatology Department of Medicine San Francisco VA Health Care System San Francisco CA USA

**Keywords:** aging, fracture healing, inflammation, macrophage, osteoimmunology, RNA‐seq

## Abstract

The elderly population suffers from higher rates of complications during fracture healing that result in increased morbidity and mortality. Inflammatory dysregulation is associated with increased age and is a contributing factor to the myriad of age‐related diseases. Therefore, we investigated age‐related changes to an important cellular regulator of inflammation, the macrophage, and the impact on fracture healing outcomes. We demonstrated that old mice (24 months) have delayed fracture healing with significantly less bone and more cartilage compared to young mice (3 months). The quantity of infiltrating macrophages into the fracture callus was similar in old and young mice. However, RNA‐seq analysis demonstrated distinct differences in the transcriptomes of macrophages derived from the fracture callus of old and young mice, with an up‐regulation of M1/pro‐inflammatory genes in macrophages from old mice as well as dysregulation of other immune‐related genes. Preventing infiltration of the fracture site by macrophages in old mice improved healing outcomes, with significantly more bone in the calluses of treated mice compared to age‐matched controls. After preventing infiltration by macrophages, the macrophages remaining within the fracture callus were collected and examined via RNA‐seq analysis, and their transcriptome resembled macrophages from young calluses. Taken together, infiltrating macrophages from old mice demonstrate detrimental age‐related changes, and depleting infiltrating macrophages can improve fracture healing in old mice.

## Graphical abstract

Delayed fracture healing in old mice is associated with transcriptomic differences between old and young macrophages infiltrating the fracture callus. An aged macrophage phenotype was characterized by increased pro‐inflammatory gene expression compared to young. Inhibition of macrophage infiltration in old mice improved fracture healing outcomes and demonstrated a resident macrophages population with a transcriptome more similar to young mice.

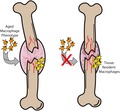

## 1 INTRODUCTION

Fracture healing follows a distinct temporal sequence characterized by an initial inflammatory phase followed by anabolic and catabolic phases (Little, Ramachandran, & Schindeler, 2007). The inflammatory phase is characterized by recruitment of innate and adaptive immune cells to the fracture site (Hankenson, Zimmerman, & Marcucio, 2014). Molecular interactions among immune cells regulate promotion and resolution of inflammation, as well as recruitment of appropriate progenitor cells necessary during the proceeding anabolic phases of fracture healing (Gerstenfeld, Cullinane, Barnes, Graves, & Einhorn, 2003). Age‐related changes in the inflammatory response and the effect on fracture healing are not well‐studied.

Perturbation of the inflammatory phase of fracture repair can have detrimental effects on the healing outcome (Hankenson et al., 2014). This is evident in patients with chronic inflammatory conditions such as diabetes, rheumatoid arthritis, and increased age. The disturbance of inflammation in these conditions is associated with poorer fracture healing outcomes (Loder, 1988; Nieminen, Nurmi, & Satokari, 1981). Similarly, in experimental animal models, local and systemic inflammatory dysregulation has negative effects on osteogenesis and fracture healing outcomes (Lin et al., 2017; Reikerås, Shegarfi, Wang, & Utvåg, 2005).

A higher incidence of bone fractures occurs in the elderly and is associated with increased rates of delayed unions and nonunions with a resulting increase in morbidity and mortality (Foulke, Kendal, Murray, & Pandit, 2016; Jones et al., 1994; Nieminen et al., 1981). Fracture models using rodents also have shown delayed healing in old mice compared to young (Lopas et al., 2014; Lu et al., 2005). Therefore, understanding how dysregulation of the inflammatory process in elderly populations affects fracture healing represents a critical area for investigation.

The elderly population, including those in good health, is found to have higher levels of circulating pro‐inflammatory cytokines, which is associated with a predisposition to a range of systemic disease including osteoporosis, Alzheimer's disease, type II diabetes, atherosclerosis, and Parkinson's disease (Giunta et al., 2008; Xia et al., 2016). The chronic, increased pro‐inflammatory status associated with aging is described as “inflamm‐aging.” Inflamm‐aging has been suggested to result from inadequate resolution of inflammation or the result of chronic stimulation that prolongs the inflammatory response (Xia et al., 2016). Currently, the mechanisms responsible for inflamm‐aging are unclear, but age‐related changes to key cellular regulators of inflammation may be responsible. We have observed increased and sustained systemic inflammation in fracture healing in old animals (Xing, Lu, Hu, Miclau, & Marcucio, 2010; Xing, Lu, Hu, Yu, et al., 2010). Further, in previous research from our laboratory, ablation of the hematopoietic stem cells of old animals via lethal irradiation, and subsequent replacement with hematopoietic stem cells of juveniles, accelerated fracture healing compared to the chimeras that received old bone marrow (Xing, Lu, Hu, Miclau, et al., 2010; Xing, Lu, Hu, Yu, et al., 2010). Thus, manipulating the inflammatory system in old animals could affect the rate of fracture healing.

The macrophage is an important inflammatory cell involved in fracture healing (Alexander et al., 2011). Throughout the course of healing, macrophages polarize among various states of inflammation in response to their environment. During early phases of healing, macrophages are classically activated and exhibit pro‐inflammatory activities. These have been generally considered M1 macrophages (Ferrante & Leibovich, 2012; Wynn, Chawla, & Pollard, 2013). As healing progresses, macrophages switch to anti‐inflammatory states and are generally considered M2 macrophages that are responsible for down‐regulating inflammation and promoting healing (Ferrante & Leibovich, 2012). However, these are very broad categories and not strict definitions of cell types, and in actuality, these categories are comprised of multiple subsets of macrophages comprising these populations. Nonetheless, enhancement of M2‐like macrophages at the fracture site has been shown to improve fracture repair (Schlundt et al., 2018). In addition, tissue‐resident macrophages, osteomacs, have been observed in close proximity to osteoblasts on the bone surface, and contribute to osteoblast regulatory functions (Chang et al., 2008). Macrophages promote osteoblast differentiation during fracture healing (Loi et al., 2016; Vi et al., 2015). We have also shown the importance of macrophages in fracture healing in previous work, where young adult mice lacking the *C‐C Motif Chemokine Receptor 2* (*Ccr2)* demonstrate significantly reduced trafficking of macrophages to the fracture callus and a resulting delay in fracture healing (Xing, Lu, Hu, Miclau, et al., 2010; Xing, Lu, Hu, Yu, et al., 2010). Others have observed similar disruption of fracture healing after depletion of macrophages in mouse models (Alexander et al., 2011; Schlundt et al., 2018; Vi et al., 2015). Finally, age‐related changes to macrophage activity have been previously demonstrated. Dysregulated chemokine and cytokine expression has been observed in aged macrophages compared to young (Gibon et al., 2016). Additionally, decreased growth factor production is associated with aged macrophages (Danon, Kowatch, & Roth, 1989). Such age‐related changes could significantly impact fracture healing in aged animals.

In this work, our goal was to better understand the contribution of the inflammatory response in aged animals, and specifically the macrophage, during fracture healing. We assessed influx of inflammatory cells to the fracture site in young and elderly mice and used next‐generation RNA sequencing to assess age‐related changes in the transcriptome of macrophages derived from the fracture callus. Finally, we manipulated macrophages in elderly mice to assess the extent to which they can be targeted for therapy. Our results add to the increasing body of evidence supporting a role for the inflammatory system in bone fracture healing.

## 2 RESULTS

### 2.1 Old mice demonstrate delayed fracture healing compared to young mice

**Figure 1:**
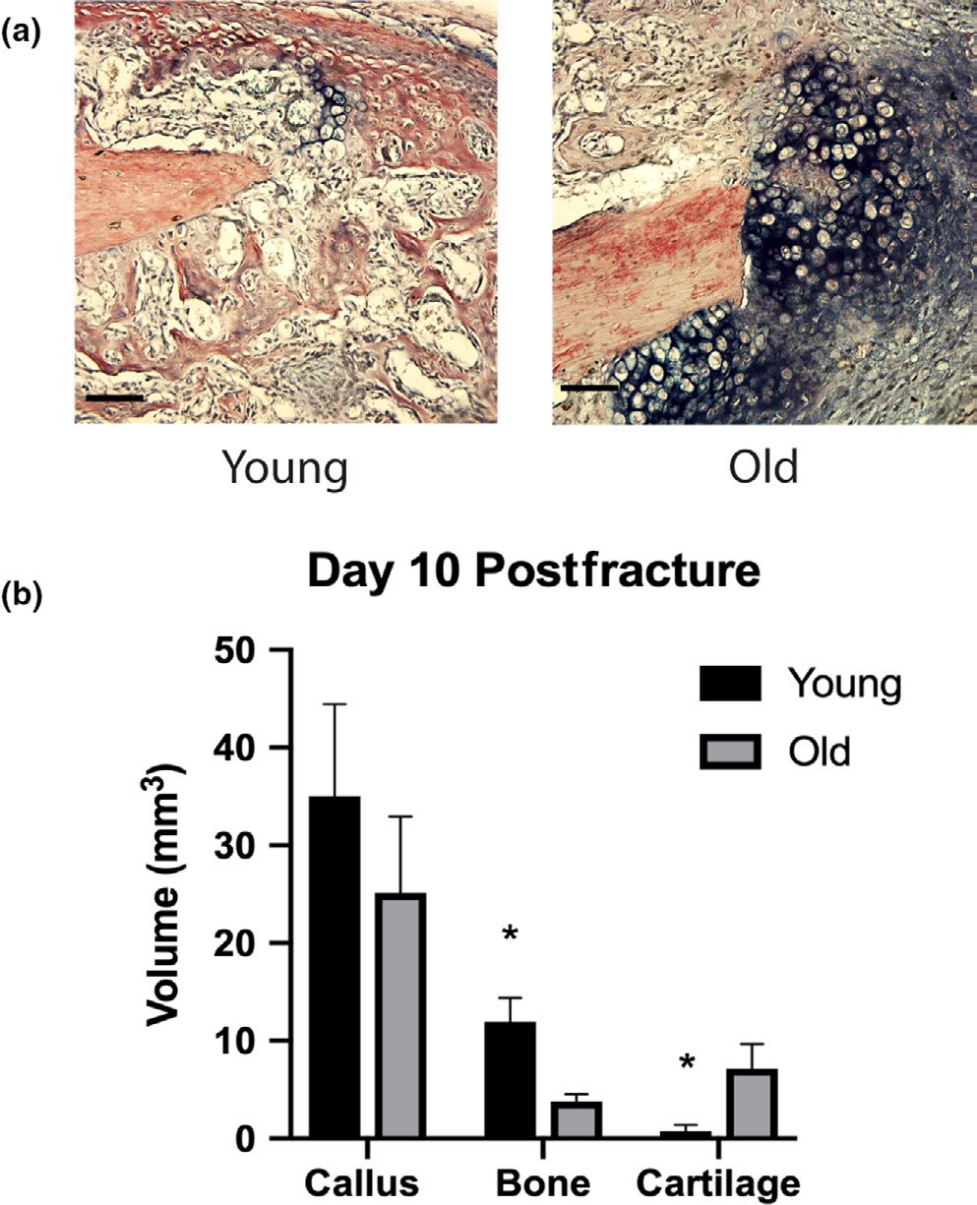
Old mice demonstrate delayed fracture healing compared to young. (a) Representative histological images (HBQ stain) of fracture calluses in old (24 months) and young (3 months) mice (scale bar = 200 µm). Stereological analysis was performed, and the volume of the total callus and the volume of bone and cartilage tissue within the callus were calculated at 10 days after closed tibial fracture (*n* = 5/group). (b) Old mice demonstrate delayed healing with smaller callus size and significantly less bone and more cartilage (*p < .05)

While there is a consensus in the field that aging impairs fracture healing, to ensure rigor and demonstrate replicability of healing outcomes, we began by comparing fracture healing in 24‐month‐old mice to young adult mice (3 months old). Twenty‐four‐month‐old mice were used because these are considered elderly (Flurkey, Currer, & Harrison, 2007). Fracture healing was assessed via stereology to quantify the volume of bone and cartilage within the fracture callus. At 10 days postfracture, old mice had smaller calluses with significantly less bone and more cartilage (*p* < .05) compared to young adult mice (Figure 1). Thus, these data are in agreement with our earlier work that demonstrated the rate of fracture healing is directly related to the age of mice with eighteen‐month‐old mice healed slower than 1‐ and 6‐month‐old mice (Lu et al., 2005).

### 2.2 Immune cell infiltration into the fracture callus is similar in old and young mice

**Figure 2:**
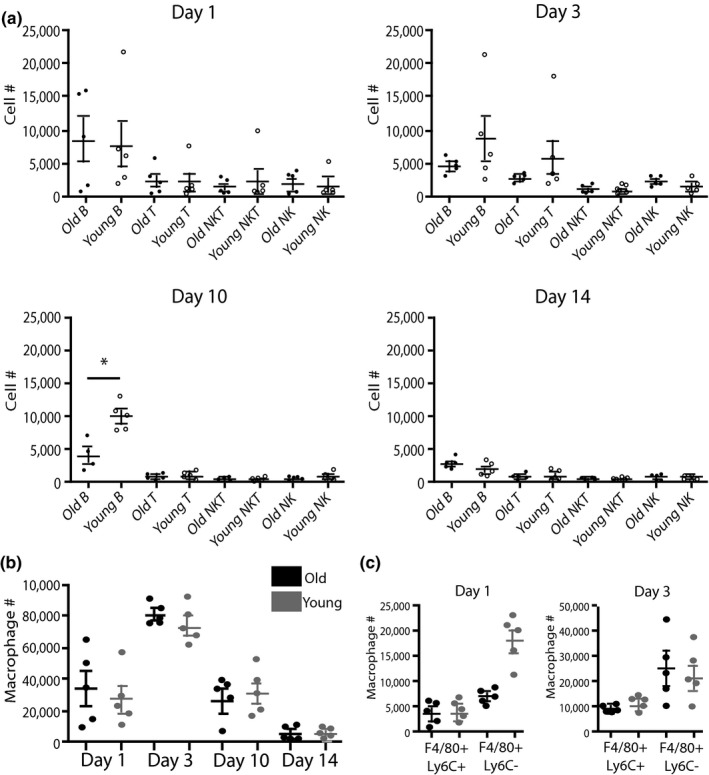
Immune cell infiltration into the fracture callus is similar in old and young mice. (a) The quantity of B cells, T cells, NKT cells, and NK cells was similar within the fracture callus at days 1, 3, 10, and 14 postfracture measured via flow cytometry in old (*n* = 5) and young (*n* = 5) mice. B‐cell quantity at day 10 was the only significant difference between age groups. (b) Macrophages (F4/80+) were the most abundant immune cell analyzed within the fracture callus and demonstrated no significant difference in quantity between young (gray) and old (black) mice at any of the time points analyzed. (c) A subpopulation of macrophages (F4/80+, Ly6C−) was increased in young mice at day 1 compared to old mice (*p < .005)

Our objective was to examine the effect of age on inflammation during fracture healing. First, we assessed the inflammatory response during fracture healing in old and young mice by quantifying lymphocyte infiltration into the fracture callus at 1, 3, 10, and 14 days postfracture via flow cytometry. The quantity of T cells, natural killer T cells, and natural killer cells isolated from the callus was similar in young and old mice at all time points examined (*n* = 5 mice/ group) (Figure 2a). In contrast, B cells were significantly increased in young mice at day 10 (*p* < .05). Macrophages were the most prevalent immune cell analyzed among cells derived from the fracture callus. The quantity of macrophages peaked 3 days after fracture, and the macrophages were reduced dramatically by day 14, but no significant differences were noted in the quantity of F4/80 + macrophages in old and young fracture calluses at any time point analyzed (Figure 2b). However, in examining subpopulations of macrophages, the F4/80+, Ly6C‐ population, indicative of a “restorative” macrophage phenotype (Ramachandran et al., 2012) was increased within the fracture callus of young mice compared to old mice at day 1, but this difference had resolved by day 3 (Figure 2c). Our data suggest that there may be slight differences in the cellular inflammatory response in young and old mice, but the differences are subtle.

### 2.3 Callus macrophages from old mice are transcriptionally distinct from callus macrophages from young mice

**Figure 3:**
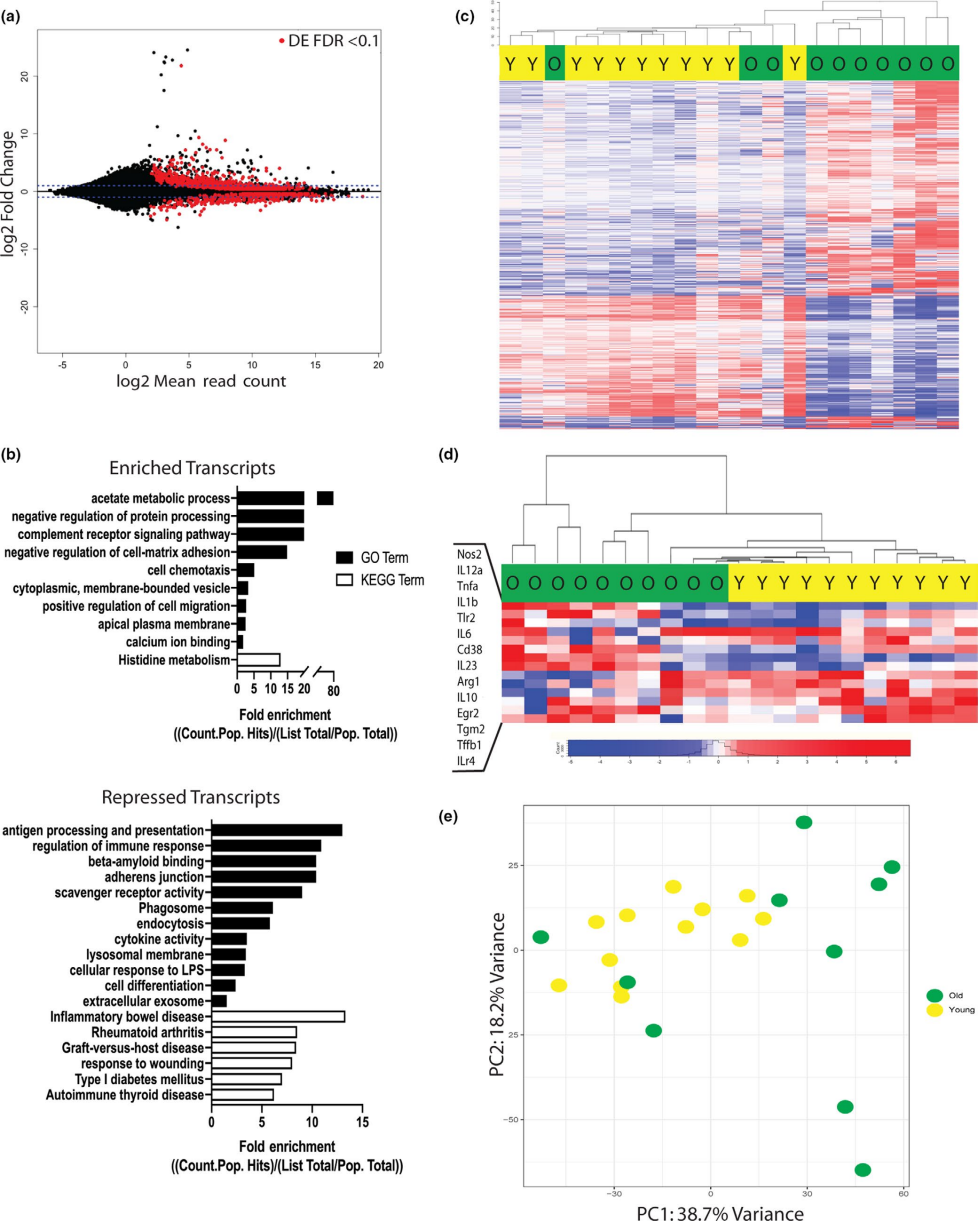
Macrophages from the fracture callus of old mice are transcriptionally distinct from young mice. RNA‐seq analysis of callus macrophages in old (*n* = 10) and young (*n* = 11) mice collected at 3 days postfracture. (a) A total of 1,222 genes were significantly differentially expressed in old macrophages compared to young (FDR < 0.1) (red dots). (b) Enriched gene ontology terms associated with the significantly up‐ and down‐regulated transcripts in macrophages from old mice compared to young. (c) Heat map demonstrates unsupervised hierarchical clustering of young (yellow) and old (green) mice based on the differential expression of the 1,222 genes. (d) Hierarchical clustering of young (yellow) and old (green) mice, based on the differential expression of a M1/M2 gene signature, demonstrates a more pro‐inflammatory/M1 gene expression signature in macrophages from old mice compared to young. (e) Principal component analysis of differential gene expression in macrophages from old and young mice demonstrates clustering of young (yellow) mice and a heterogenous spread of old (green) mice across PC1 and PC2

Since the quantity of immune cells infiltrating the fracture callus was similar in young and old mice, functional, rather than quantitative, changes in these cells may contribute to inflammatory dysregulation upon aging. The macrophage was selected for further analysis, because macrophages were the most abundant immune cell type analyzed, and we observed differences in a subpopulation of macrophages, F4/80+, Ly6C‐, between young and old mice. To evaluate intrinsic age‐related changes in macrophages, RNA‐seq analysis was performed on F4/80 + macrophages isolated from the fracture callus of old and young mice at 3 days postfracture. In total, 1,222 genes were significantly differentially expressed in old macrophages compared to young; 364 genes were up‐regulated and 200 genes were down‐regulated more than twofold (Figure 3a). Gene ontology enrichment analysis was performed to begin exploring the implications of these differentially expressed genes in macrophages (Figure 3b). A number of significantly enriched disease processes are identified that are related to aging and the immune response. These enriched terms include rheumatoid arthritis, graft‐versus‐host disease, and inflammatory bowel disease. Additionally, molecular and cellular processes important in macrophage function were significantly enriched, including antigen processing and presentation, response to wounding, and cytokine activity.

The 1,222 significantly differentially expressed genes in the macrophages from old and young fracture calluses are compared with a heat map (Figure 3c). Unsupervised hierarchical clustering based on Euclidean distances demonstrates old and young mice sort largely based upon differential gene expression patterns (Figure 3c). The heatmap further characterizes old mice as more heterogeneous in their transcriptomes than young mice. To further assess differences between old and young macrophages, samples were hierarchically sorted based on their differential expression of 14 genes associated with characteristic macrophage cytokines and markers of M1 and M2 macrophages. This analysis demonstrated that mice sort by age and that old mice have increased expression of pro‐inflammatory cytokines and markers of M1 macrophages (Figure 3d). Principal component analysis further demonstrates distinct clustering of the young mice from old (Figure 3e). The heterogeneity of the old macrophage transcriptome is further seen in the principal component analysis with young mice demonstrating closer clustering compared to old mice that span PC1 and PC2 (Figure 3e). Although three old mice are seen clustering with the young mice in all analyses performed, this further supports the idea that cells from old mice are more variable in their gene expression. PC1 and PC2 account for 56.9% of the variance, and mean PC scores of old and young mice across PC1 and PC2 additionally demonstrate significant differences by age and the increased variability in the old mice (Figure S1). PCs 1–5 accounted for 76.5% of the variance, and independent group analysis demonstrated the mean PC scores of PCs 1–5 were significantly different in old mice compared to young via Hotelling's T‐squared test (*T* ^2^ = 20.04, *df* = 5,15, *p* = .04). As PC1 appears to largely separate the old from young mice, transcripts with the highest and lowest eigenvector coefficients on PC1 are presented in Table S1 to show the list of genes that most strongly contribute to the separation of old and young macrophages along PC1.

### 2.4 Inhibition of macrophage recruitment improves fracture healing in old mice

**Figure 4:**
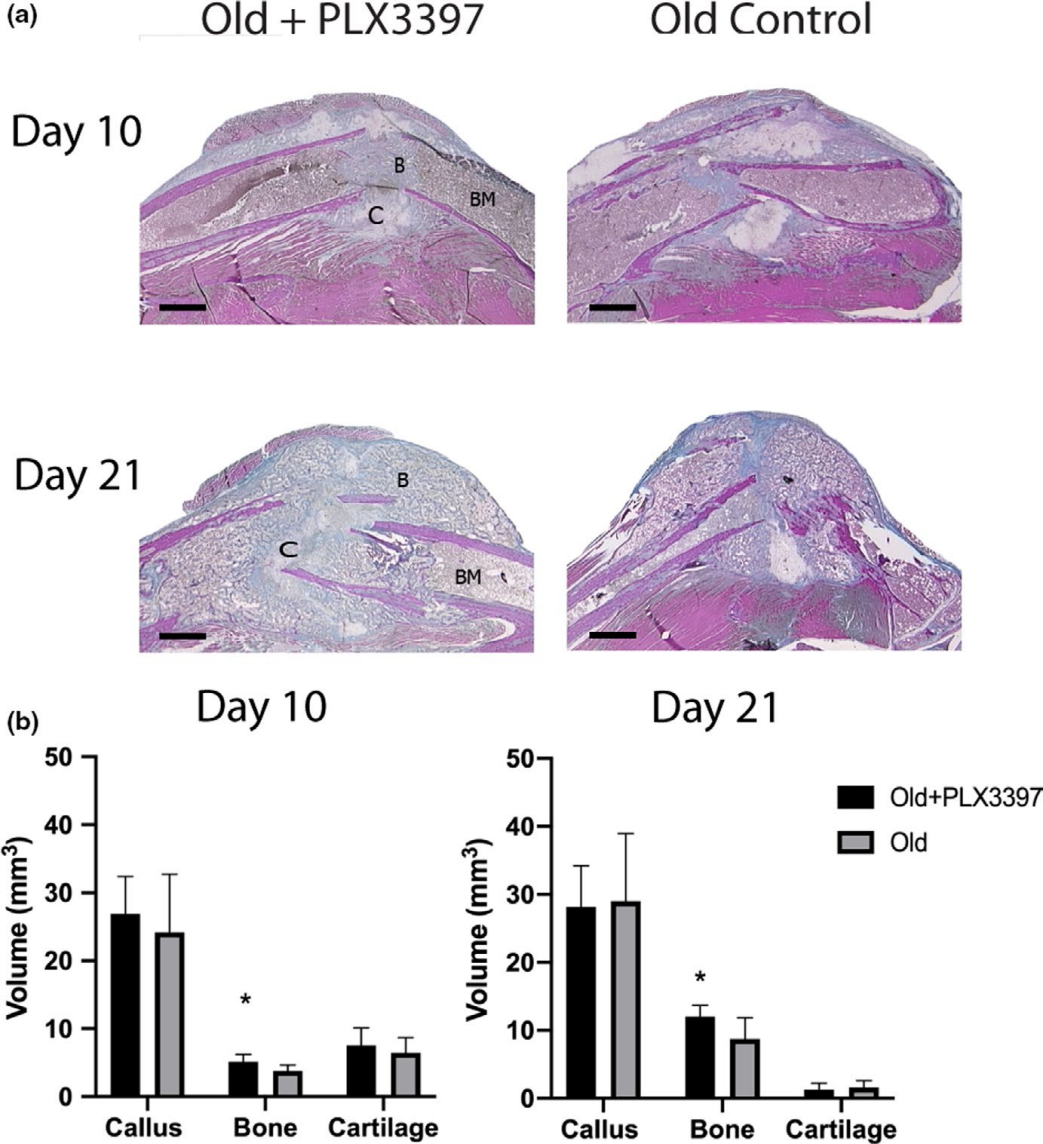
Inhibition of macrophage recruitment improves fracture healing in old mice. (a) Representative histological images (modified Milligan's trichrome stain) of fracture calluses in old mice treated with PLX3397 and age‐matched old controls 10 days and 21 days after closed tibial fracture (*n* = 6/group) (scale bar = 1mm). B—bone; C—cartilage; BM—bone marrow. Stereological analysis was performed, and the volume of the total callus and the volume of bone and cartilage tissue within the callus were calculated. (b) Old mice treated with PLX3397 demonstrate significantly more bone volume compared to old nontreated mice at both time points (*p < .05)

**Figure 5:**
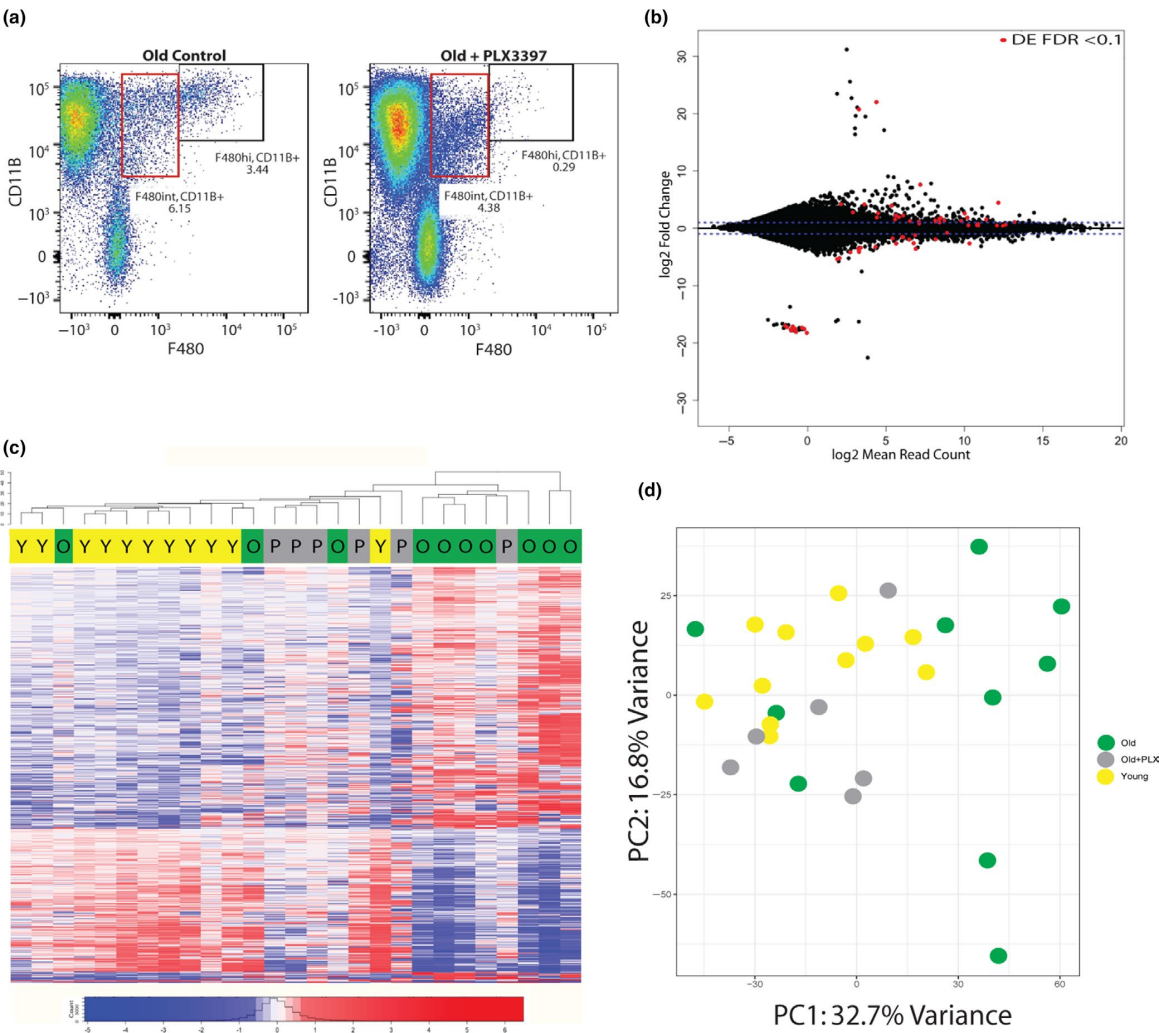
Improved fracture healing in treated old mice is associated with transcriptionally “younger” macrophages. (a) PLX3397 treatment during fracture healing resulted in significant decrease in macrophage quantity within the fracture callus, as analyzed through flow cytometry. (b) RNA‐seq analysis demonstrates 64 genes were significantly differentially expressed in old macrophages treated with PLX3397 compared to young (red dots) (FDR < 0.1). (c) Hierarchal clustering demonstrates old macrophages treated with PLX3397 (gray) cluster between young (yellow) and old (green) control macrophages. (d) Principal component analysis of differential gene expression in macrophages demonstrates close clustering of young (yellow) mice with old mice treated with PLX3397 (gray)

Macrophages from the fracture calluses of old mice were transcriptionally distinct and displayed a more pro‐inflammatory phenotype compared to young macrophages. Thus, we sought to inhibit macrophage recruitment during fracture healing to assess whether healing outcomes could be improved in old mice. We administered a M‐CSF‐1R inhibitor, PLX3397 (Pexidartinib, Plexxikon, CA, USA), that inhibits recruitment of macrophages from the bone marrow. Administering PLX3397 for 10 days and 21 days after fracture healing improved fracture healing outcomes (Figure 4). Stereological analysis demonstrated a larger fracture callus with significantly increased bone volume in treated old mice compared to control old mice at both time points (Figure 4b). Flow cytometry demonstrated significant reduction of macrophages within the callus of PLX3397‐treated mice (Figure 5a). Administration of PLX3397 during fracture healing in young mice demonstrated no effect at day 10 postfracture (Figure S2).

### 2.5 Improved fracture healing is associated with transcriptionally “younger” macrophages

To understand how the transcriptional profile of callus macrophages changes with PLX3397 treatment, we collected macrophages from the fracture callus of mice treated with PLX3397 at 3 days postfracture. RNA‐seq analysis demonstrated that only 64 genes were significantly differentially expressed in old macrophages from mice treated with PLX3397 compared to young macrophages; 27 genes were up‐regulated and 28 genes were down‐regulated more than twofold (Figure 5b). Unsupervised hierarchical clustering by Euclidean distance demonstrates that macrophages from old mice treated with PLX3397 cluster between macrophages from young and old mice without treatment (Figure 5c). Principal component analysis further demonstrates that the macrophages from old mice treated with PLX3397 cluster closely with the young mice compared to the old with less transcriptomic heterogeneity (Figure 5d). This suggests that the inflammatory macrophages that are recruited to the bone fracture are substantially different between old and young mice, but the remaining tissue‐resident macrophages demonstrate less age‐related changes.

## 3 DISCUSSION

The results from this study demonstrate that an aged macrophage phenotype is detrimental to fracture healing. Using an unbiased next‐generation sequencing approach, we demonstrate differences in the gene expression signatures of macrophages that infiltrated the fracture site in young and old mice. Macrophages from old mice have a more M1, pro‐inflammatory gene signature than macrophages from young animals. Further, we demonstrated that a pharmacologic (PLX3397) leading to a decrease in macrophages recruitment to the fracture site of old mice improves fracture healing outcomes. In older mice treated with PLX3397, macrophages that are present within the callus appear transcriptionally “younger,” suggesting that the detrimental age‐related changes may occur in infiltrating macrophages.

The delayed fracture healing we observed in the elderly mice compared to young adults here (Figure 1) is similar to our previous findings (Lu et al., 2005). Other groups have shown delayed healing with decreased callus size and decreased bone volume at multiple time points postfracture in old mice compared to young (Lopas et al., 2014; Meyer et al., 2003). Substantial alterations in inflammation may affect fracture healing in aged animals. For example, inflammation induced with lipopolysaccharide led to decreased callus strength in young animals (Reikerås et al., 2005). Also, delayed healing in aged animals has been directly associated with inflammatory dysregulation within the callus (Hebb et al., 2018; Xing, Lu, Hu, Miclau, et al., 2010; Xing, Lu, Hu, Yu, et al., 2010). Other studies have demonstrated an association of systemic inflammatory dysregulation, as a result of increased age or disease, with poor fracture healing outcomes in humans and animal experiments (Clark, Nakamura, Miclau, Marcucio, & Marcucio Ralph, 2017; Loder, 1988). In fact, we have shown that transplantation of juvenile bone marrow into lethally irradiated middle‐aged animals stimulates bone fracture healing, and this is associated with decreased inflammation (Xing, Lu, Hu, Miclau, et al., 2010; Xing, Lu, Hu, Yu, et al., 2010). This work was subsequently confirmed (Baht et al., 2015). However, the inflammatory cells responsible for the stimulatory effect remain largely unknown.

Recent work has suggested that macrophages may underlie the dysregulation of inflammation seen during fracture healing in aged animals. Our preliminary research demonstrated that reducing the influx of inflammatory macrophages into the callus of aged animals stimulated healing (Slade Shantz, Yu, Andres, Miclau, & Marcucio, 2014). Other work has advanced this observation and supports the idea that aging macrophages are deleterious to healing (Baht et al., 2015; Xing, Lu, Hu, Miclau, et al., 2010; Xing, Lu, Hu, Yu, et al., 2010). Collectively, this work supports the important role for macrophages in fracture healing and the deleterious effect of age‐related changes to macrophages on fracture healing outcomes.

The age‐related changes in fracture healing do not appear to be a function of significant differential inflammatory cell recruitment. We observed similar numbers of immune cells infiltrating the fracture callus in young and old mice (Figure 2). T cells may contribute to fracture healing largely through the recruitment and activation of osteoclasts (Baht, Vi, & Alman, 2018). The aging immune system is characterized by decreased naive T‐cell quantity and weaker activation in elderly populations compared to young (Desai, Grolleau‐Julius, & Yung, 2010). However, the quantity of T cells within the fracture callus did not differ by age (Figure 2a). Similarly, the quantity of F4/80 + macrophages did not differ by age; however, a subpopulation of macrophages, identified as F480 + Ly6C−, was increased at day 1 postfracture in young animals but this difference was not apparent by day 3 (Figure 2c). Macrophages that are F4/80‐positive and Ly6C‐negative have been suggested to be anti‐inflammatory or M2‐like mice to a greater extent at earlier time points than in old mice. The significance of this subtle alteration is not known.

At day 10, we observed increased B cells in the fracture callus of young mice compared to old (Figure 2a). B cells have demonstrated age‐related changes that affect their function (Miller & Cancro, 2007). However, the contribution of B cells to fracture healing is largely unknown. During fracture healing, B cells regulate osteoclast activity through the expression of osteoprotegerin (Baht et al., 2018), and interactions between B cells and macrophages have been demonstrated to regulate inflammation during infection (Harvey, Gee, Haberman, Shlomchik, & Mamula, 2007). While these are intriguing observations, the specific role of B cells in mediating effects of age on fracture healing is unexplored.

The importance of macrophages to fracture healing and bone regeneration is beginning to emerge (Alexander et al., 2011; Schlundt et al., 2018; Xing, Lu, Hu, Miclau, et al., 2010; Xing, Lu, Hu, Yu, et al., 2010). Different macrophage sub‐types have been proposed to mediate separate functions during healing. Pro‐inflammatory, or M1‐type, macrophages appear early in fracture healing and produce pro‐inflammatory cytokines (IL‐1, IL‐6, TNFα, iNOS) that further propagate the inflammatory response (Wynn et al., 2013). Later, a switch to anti‐inflammatory, or M2‐type, macrophages occurs within the fracture callus. M2 macrophages initiate down‐regulation of the inflammatory response with production of IL‐10 and TGFβ (Ferrante & Leibovich, 2012). M2 macrophages also promote tissue repair through the production of growth factors (TGFβ, PDGF, VEGF) (Wynn et al., 2013). However, in vivo phenotyping of macrophages is complex, and given the plasticity of macrophages, the M1/M2 distinctions are likely not dichotomous and probably represent poles on a large spectrum of macrophage phenotypes. Here, RNA‐seq analysis allowed an unbiased analysis of the transcriptomic differences of macrophages within the fracture callus of young and old mice. Macrophages from the fracture callus of old mice were transcriptionally distinct from macrophages of young mice (Figure 3). Further, a M1/M2 gene expression signature, comprised of 14 selected genes for cytokines and cell markers associated with traditional M1 and M2 phenotypes, was distinct between age groups and able to differentiate macrophages from old mice versus young. Here, macrophages from old mice demonstrated expression of more pro‐inflammatory or M1 genes suggesting that old macrophages contribute to the pro‐inflammatory phenotype evident in elderly populations.

With the demonstrated pro‐inflammatory, and potentially deleterious, phenotype of aged macrophages, we wanted to understand the effect of limiting macrophage recruitment into the fracture callus. PLX3397 treatment prevented macrophage recruitment and resulted in improved fracture healing in old mice (Figure 4). The magnitude of change appears small in the PLX3397‐treated old mice when compared to total callus volume. However, the change represents an approximately 35% increase in bone volume in the treated groups compared to age‐matched controls. The increased bone volume is consistent with other work in mice that shows improvements in bone volume within a fracture callus of 25%–50% using other experimental agents to improve fracture healing (Shen et al., 2009; Street et al., 2002). Here, we analyzed the improved healing within the early callus. Mechanical testing of the bone at later time points could demonstrate additional differences as a function of age. However, later stages of healing incorporate processes that do not rely on the activity of macrophages and are thus outside the scope of this work.

RNA‐seq analysis demonstrated that 1,222 genes were significantly differentially expressed in macrophages in old mice compared to young (Figure 3a), and this quantity is important considering the breadth of biological and disease processes that these genes are associated with (Figure 3b). In old mice, when recruitment of macrophages was inhibited with PLX3397, the number of significantly differentially expressed genes between old and young macrophages was reduced by 95% (Figure 5b), and the treated old mice clustered between the young and old controls (Figure 5c). These findings suggest the presence of a more youthful macrophage population after PLX3397 treatment in old mice.

One potential “youthful” macrophage population is osteomacs. Osteomacs are tissue‐resident macrophages in bone and have been shown to co‐localize with osteoblasts and contribute to osteogenesis (Chang et al., 2008; Vi et al., 2015). Depletion of osteomac populations in vivo was shown to be deleterious during both intramembranous and endochondral fracture repair processes (Batoon et al., 2019). Here, the pharmacological effect of PLX3397 works largely on infiltrating inflammatory macrophages by antagonizing M‐CSF1R and preventing the monocyte‐to‐macrophage differentiation. Therefore, we suspect that the resident osteomacs are less affected by PLX3397. The improved fracture healing in old mice treated with PLX3397 could be a result of decreased inflammatory macrophages and/or an expansion or activation of a more youthful and beneficial osteomac population. Further work is needed to understand the age‐related changes to osteomacs and their contribution to fracture healing.

A potentially important observation made was that macrophages from old animals were more heterogenous than those from young animals (Figure 3). The extent of heterogeneity in old mice is present despite all mice being from the same genetic background, sourced from the same laboratory and colony, housed in similar environments, and samples prepared on the same day. Complex disease processes and biological traits, including aging, are often defined by a heterogenous phenotypic presentation (Cannon et al., 2017; Muller‐Sieburg, Sieburg, Bernitz, & Cattarossi, 2012). Heterogenous changes are present across many aspects of the biology of aging with an impact that is not fully understood. Age‐related genetic heterogenicity could result from cumulative effects from the environment or an unknown mechanism (Kulminski et al., 2018). How to properly analyze the heterogenicity present in large genetic datasets is not clearly defined. Largely, the heterogeneity is accepted as normal and sample size is increased so that differences may be detected. However, further research would be better aimed at understanding the biological cause and significance of such variation, as the increased variance in old animals may be a substantial contributor to phenotypic outcomes. Further, this may aid in identifying at‐risk individuals who would benefit from individualized treatment plans.

In conclusion, this study characterizes the cellular immune response during bone fracture healing and demonstrates age‐related changes at the cellular level that are reflective of the altered physiology present in elderly populations. Robust transcriptional differences differentiated macrophages infiltrating the callus of old mice compared to young. The old macrophages demonstrated a pro‐inflammatory M1 macrophage phenotype. The aged macrophage phenotype was detrimental to fracture healing outcomes as fracture healing was improved in old mice when aged macrophages were inhibited from accumulating in the callus during fracture healing. The therapeutic targeting of macrophages during fracture healing may be an effective therapy to improve fracture healing outcomes in elderly populations. Finally, understanding the variance and its underlying mechanism(s) may contribute significantly to directed treatments of the patient with musculoskeletal injuries.

## 4 EXPERIMENTAL PROCEDURES

### 4.1 Animals

All procedures were approved by the UCSF Institutional Animal Care and Use Committee and conducted in accordance with the National Institutes of Health guidelines for humane animal care. All mice (C57B6/J) were obtained from the National Institute on Aging's Aged Rodent Colony. Specific pathogen‐free mice were bred and raised in barriers and utilized for experiments at 24 months (old adult mice) or 3 months (young adult mice) of age.

### 4.2 Tibia fractures

Mice were anesthetized and subjected to closed, nonstable fractures of the right tibia created by three‐point bending, as previously described (Xing, Lu, Hu, Miclau, et al., 2010; Xing, Lu, Hu, Yu, et al., 2010). Analgesics were administered postsurgery, and mice were permitted to ambulate freely. To inhibit macrophage recruitment during fracture healing in old mice, treatment groups received the compound PLX3397 (275 mg/kg) (Plexxikon Inc. Berkeley, CA) ad libitum in their chow. Control groups received the same chow without PLX3397. PLX3397 is a small‐molecule kinase inhibitor of colony‐stimulating factor 1 receptor (M‐CSF1R) (Butowski et al., 2016). The compound significantly reduces the quantity of macrophages in the fracture callus (Slade Shantz et al., 2014). Treatment with PLX3397 was started 24 hr before fracture and continued for 3, 10, or 21 days after fracture.

### 4.3 Tibia processing and stereology

Mice were sacrificed at day 10 or 21 postfracture for stereological analysis of the fracture callus. Fractured tibiae were collected and fixed for 24 hr in 4% paraformaldehyde. The tibiae were decalcified in 19% EDTA for 14 days and dehydrated in graded ethanol prior to paraffin embedding. Serial sagittal sections (10 μm) were cut through the entire tibia using a microtome (Lecia, Bannockburn, IL) and mounted on slides. Sections were stained using Hall–Brunt Quadruple Stain (HBQ) to visualize bone and cartilage. An Olympus CAST system (Center Valley, PA) and software by Visiopharm (Hørsholm, Denmark) was used to quantify tissue volumes according to stereological methods developed by Howard and Reed (Howard & Reed, 1998). A 2x magnification setting was utilized to outline the boundary of the fracture callus. Then, bone and cartilage were identified and labeled at 20x magnification. The Cavalieri formula was used to estimate the absolute volume of the total callus, bone, and cartilage tissue as previously described (Lu et al., 2005; Xing, Lu, Hu, Miclau, et al., 2010; Xing, Lu, Hu, Yu, et al., 2010).

### 4.4 Flow cytometry

Old and young mice were sacrificed at days 1, 3, 10, and 14 postfracture. Fractured tibiae were collected, and the calluses were dissected, weighed, disassociated manually through a 100‐µm nylon cell strainer, and digested with collagenase type I (0.2 mg/ml; Worthington, Lakewood, NJ) for 1 hr at 37 degrees. Cells were rinsed, collected by centrifugation, and resuspended in incubation buffer (0.5% BSA in PBS). Isolated cells were blocked for 10 min at room temperature in 10% rat serum and then stained with directly conjugated fluorescent antibodies: CD45 (clone 30‐F11), CD3 (145‐2C11), B220 (RA3682), Gr‐1 (RB6‐8C5), NK1.1 (PK136), MHC Class II (M5/114.15.2), Ly6C (HK1.4), Ly6G (clone 1A8), F4/80 (clone BM8), and CD11b (clone M1/70) (Biolegend, San Diego, CA). Staining with Fixable Red Dead (Thermo Fisher, Waltham, MA) was used for the detection of dead cells. Isotype controls and fluorescence minus one controls were used to gate for background staining. Cells were analyzed and/or sorted on a FACSAria (BD Biosciences, San Jose, CA). FlowJo Software 9.6 (Treestar, Ashland, OR) was used for analysis.

### 4.5 RNA‐seq analysis

Macrophages were isolated from the fracture callus of old (*n* = 10), old treated with PLX3397 (*n* = 6) and young (*n* = 11) mice at day 3 postfracture. The callus was dissected, and cells were collected as described above. For the detection and isolation of macrophages, cells that stained with the following directly conjugated fluorescent antibodies CD3 (145‐2C11), B220 (RA3682), NK1.1 (PK136), and Ly6G (clone 1A8) were excluded, and macrophages were collected by staining with CD45 (clone 30‐F11), F4/80 (clone BM8), and CD11b (clone M1/70) (Biolegend, San Diego, CA). Callus macrophages were sorted to 99.8% purity on FACSAria (BD Biosciences, San Jose, CA). RNA was extracted using Invitrogen RNA aqueous Micro Kit (AM1931). The library was prepared using Illumina TruSeq Stranded mRNA Library Prep Kit, and single‐end 50 bp RNA‐seq was performed on Illumina HiSeq 4,000. An average read depth of 60.7 million reads per sample was generated. Reads were aligned using STAR_2.4.2a to the mouse genome (Ensemble Mouse GRCm38.78). Differential gene expression was assessed using DEseq2. Gene ontology and KEGG pathway analysis was performed using DAVID (http://david.abcc.ncifcrf.gov), and the mouse genome was used as background.

### 4.6 Statistics

GraphPad Prism v.7 software was used for analysis. Comparisons between groups were made by first using a 2‐way ANOVA multiple comparisons test followed by a 2‐tailed Student's *t* test. *p* < .05 was considered statistically significant. Differential gene expression was considered significant at FDR < 0.1. For term enrichment in gene ontology and KEGG pathway analysis, the level of significance was set using a modified Fisher's exact P‐value of *p* < .05.

## CONFLICT OF INTEREST

Theodore Miclau has acted as a paid consultant for the following; Amgen, Bone Therapeutics, Arquos, Surrozen, and DePuy, and has received financial or material support from the following: Baxter, Inman Abbott Society, Orthopaedic Research Society, International Combined Orthopaedic Research Societies, Osteosynthesis and Trauma Care Foundation, and AO Foundation/AO Research Institute Advisory Committee. We have obtained an M‐CSFR inhibitor (PLX3397) from Plexxikon Inc. (Berkeley, CA) for this research. All other authors report no conflict of interest.

## AUTHOR CONTRIBUTIONS

Daniel Clark, Mary Nakamura, and Ralph Marcucio conceptualized the study. Daniel Clark, Sloane Brazina, Frank Yang, Diane Hu, Christine Hsieh, and Erene Niemi contributed to methodology. Daniel Clark, Sloane Brazina, Christine Hsieh, Mary Nakamura, and Ralph Marcucio involved in formal analysis. Daniel Clark, Sloane Brazina, Frank Yang, Diane Hu, Christine Hsieh, and Erene Niemi investigated the study. Daniel Clark wrote the original draft of the manuscript and visualized the data. Sloane Brazina validated the study. Theodore Miclau, Mary Nakamura, and Ralph Marcucio provided resources and acquired funding. Theodore Miclau and Ralph Marcucio reviewed and edited the manuscript. Theodore Miclau supervised the study. Ralph Marcucio administrated the project.

## Data Availability

The data that support the findings of this study are openly available in figshare at http://doi.org/10.6084/m9.figshare.9275000
