# Supplementary material for: Age‐related changes to macrophages are detrimental to fracture healing in mice
Source: Aging Cell. 2020 Feb 25;19(3):e13112. doi: 10.1111/acel.13112 (PMC7059136; doi:10.1111/acel.13112)
Supplement: Supplementary file 1 [file ACEL-19-e13112-s001.pdf]

Supporting Information

A.

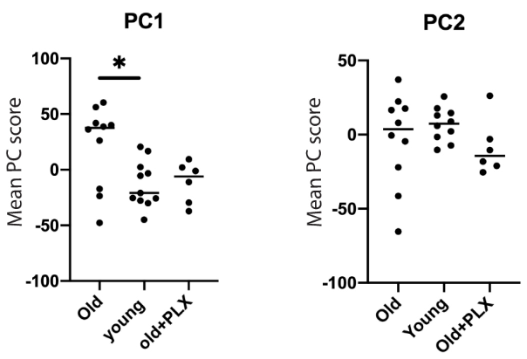

B.

Old vs Young		
	% variance explained	t test (p value)
PC1	38.7	0.02
PC2	18.2	0.35
PC3	8.9	0.02
PC4	6.2	0.23
PC5	4.5	0.94

C.

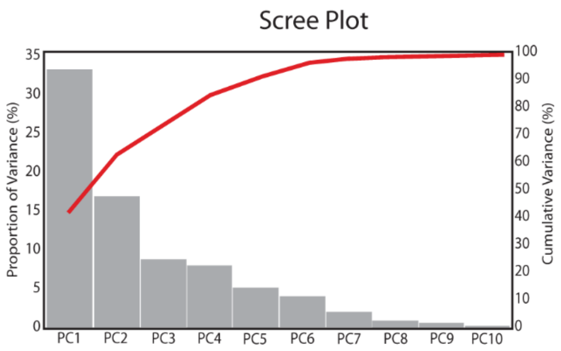

Supplemental Figure 1: (A) Plot of the individual mean PC scores along PC1 and PC2 of young, old, and old mice treated with PLX3397. (B) The mean PC scores of PC1 and PC3 were significantly different in macrophages from old mice compared to young. (C) Scree plot demonstrates that the majority of the variance (56.9%) is accounted for in PC1 and PC2 and 76.5% is accounted by PCs 1-5.

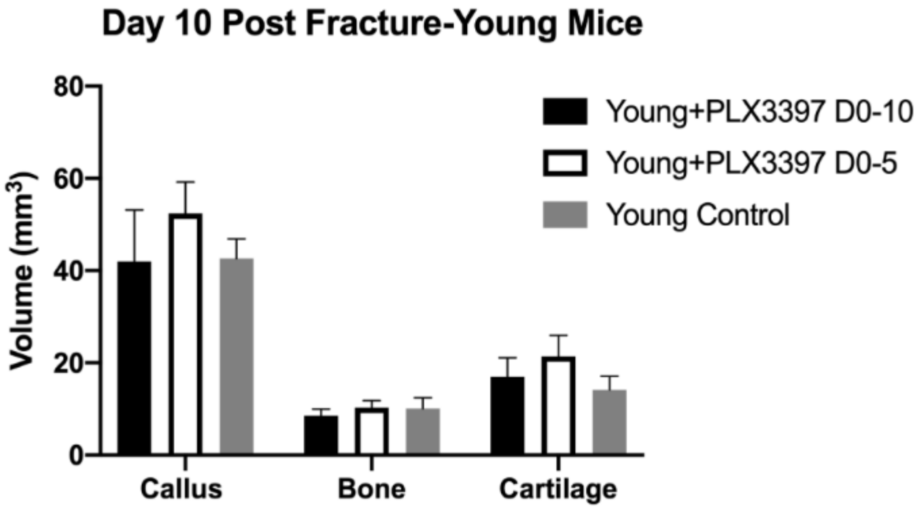

Supplemental Figure 2: Inhibition of macrophage recruitment does not affect fracture healing in young mice. PLX3397 was administered to young mice during fracture healing for the first 5 days or for the entire 10 day healing period. At 10 days post fracture the callus was isolated and stereological analysis was performed. Treatment with PLX3397 had no effect on callus, bone, or cartilage volume compared to control mice ($p>0.05$).

Supplementary Table 1: Eigenvector coefficients of the upper and lower 2% of transcripts along PC1.

Upper 2%			Lower 2%	
**Gene**	**Eigenvector**		**Gene**	**Eigenvector**
Igkv15-103	0.0584		Gpnmb	-0.0425
Ighg2c	0.0556		Selenop	-0.0426
Ighv7-1	0.0547		Slc6a8	-0.0428
Igkc	0.0537		P2ry6	-0.0429
Ighg2b	0.0531		Pdlim4	-0.0432
Igkj5	0.0439		Tppp3	-0.0436
Ighv6-3	0.0435		Lpl	-0.0437
Igkv5-39	0.0430		Fcgr1	-0.0440
Igkv1-117	0.0426		Cav1	-0.0443
BC100530	0.0426		Igf1	-0.0445
Stfa2l1	0.0425		Pid1	-0.0446
Igkv1-88	0.0418		Tmem37	-0.0447
Igkv6-32	0.0388		H2-Ab1	-0.0448
Asprv1	0.0385		Cx3cr1	-0.0449
Stfa2	0.0383		Gdf15	-0.0449
Igkj2	0.0368		Il10	-0.0449
Ighv14-2	0.0356		Mmp14	-0.0450

Aspa	0.0351		Ednrb	-0.0451
Stfa1	0.0343		H2-Aa	-0.0452
Ighv11-2	0.0343		Ifi27l2a	-0.0453
Gm5483	0.0335		C3ar1	-0.0458
Pou2af1	0.0334		H2-Eb1	-0.0461
Iglc2	0.0325		Ccr5	-0.0465
Igha	0.0323		Stab1	-0.0468
Ighj4	0.0322		Olfml3	-0.0469
Ighv5-16	0.0316		Aif1	-0.0477
Hoxa9	0.0309		Emp1	-0.0477
Ctla2a	0.0309		Amz1	-0.0482
Cacna1h	0.0307		Abcc3	-0.0483
Gm27252	0.0305		Pmp22	-0.0487
Ighm	0.0301		Apoe	-0.0489
Gimap6	0.0301		Pltp	-0.0489
D630045J12Rik	0.0298		Cd36	-0.0494
Stfa3	0.0297		Spp1	-0.0495
Il5ra	0.0296		Ccl12	-0.0497
Hoxa7	0.0288		Dab2	-0.0497
Fam110c	0.0287		Gas6	-0.0497
Saa3	0.0287		Ifi205	-0.0498
Pdgfrb	0.0286		Nxpe5	-0.0500
Dmwd	0.0284		Lgmn	-0.0507
Spns2	0.0284		Ccl8	-0.0519
Mpl	0.0279		Pf4	-0.0520
Igkj4	0.0279		Flrt3	-0.0524
Cxcr2	0.0278		C1qc	-0.0526
Ighj2	0.0277		Folr2	-0.0527
Iglv1	0.0277		Siglec1	-0.0539
Retnlg	0.0274		Ms4a14	-0.0539
Igkv5-43	0.0273		Cxcl1	-0.0545
Il17rb	0.0273		Ppbp	-0.0548
Ighv9-3	0.0270		Sdc3	-0.0551
Scrg1	0.0269		C1qb	-0.0558
Ighj1	0.0268		Cbr2	-0.0570
Ighv1-15	0.0266		Pdpn	-0.0582
Myct1	0.0265		Arg1	-0.0589
Crispld2	0.0265		C1qa	-0.0596
Muc13	0.0265		Ccl7	-0.0598
Cd55	0.0265		Cd209a	-0.0598
Epx	0.0264		Ms4a7	-0.0610
Itga2b	0.0263		Mrc1	-0.0656
Serpina3g	0.0263		Fcrls	-0.0737
